# Natural Variation in Partial Resistance to *Pseudomonas syringae* Is Controlled by Two Major QTLs in *Arabidopsis thaliana*

**DOI:** 10.1371/journal.pone.0000123

**Published:** 2006-12-27

**Authors:** Laure Perchepied, Thomas Kroj, Maurice Tronchet, Olivier Loudet, Dominique Roby

**Affiliations:** 1 Laboratoire des Interactions Plantes-Microorganismes (LIPM), UMR Centre National de la Recherche Scientifique/National Institute for Agronomical Research 2594, Castanet-Tolosan, France; 2 National Institute for Agronomical Research (INRA), Versailles, France; University of Chicago, United States of America

## Abstract

**Background:**

Low-level, partial resistance is pre-eminent in natural populations, however, the mechanisms underlying this form of resistance are still poorly understood.

**Methodology/Principal Findings:**

In the present study, we used the model pathosystem *Pseudomonas syringae* pv. *tomato* DC3000 (*Pst*) - *Arabidopsis thaliana* to study the genetic basis of this form of resistance. Phenotypic analysis of a set of Arabidopsis accessions, based on evaluation of *in planta* pathogen growth revealed extensive quantitative variation for partial resistance to *Pst*. It allowed choosing a recombinant inbred line (RIL) population derived from a cross between the accessions Bayreuth and Shahdara for quantitative genetic analysis. Experiments performed under two different environmental conditions led to the detection of two major and two minor quantitative trait loci (QTLs) governing partial resistance to *Pst* and called *PRP-Ps1* to *PRP-Ps4*. The two major QTLs, *PRP-Ps1* and *PRP-Ps2*, were confirmed in near isogenic lines (NILs), following the heterogeneous inbred families (HIFs) strategy. Analysis of marker gene expression using these HIFs indicated a negative correlation between the induced amount of transcripts of SA-dependent genes *PR1*, *ICS* and *PR5*, and the *in planta* bacterial growth in the HIF segregating at *PRP-Ps2* locus, suggesting an implication of *PRP-Ps2* in the activation of SA dependent responses.

**Conclusions/Significance:**

These results show that variation in partial resistance to *Pst* in Arabidopsis is governed by relatively few loci, and the validation of two major loci opens the way for their fine mapping and their cloning, which will improve our understanding of the molecular mechanisms underlying partial resistance.

## Introduction

Plants are exposed to a wide variety of pathogens with different invasion strategies. Successful infections are, however, relatively rare because plants have evolved powerful preformed and inducible defence mechanisms to restrict pathogen growth. Nonhost resistance relies on multiple mechanisms, which are beginning to be uncovered [1]–[3]. This defense system shows some similarity with the mammalian innate immunity [4], [5] and is associated with multiple signal transduction events like an oxidative burst, ion fluxes, activation of MAP kinase cascades, with the transcriptional induction of pathogen-responsive genes and with localized callose deposition at the cell wall [6]–[9]. If a pathogen can overcome nonhost resistance, it can spread in its host plant; however, different defense mechanisms in plants can still be activated, leading to complete or partial resistance. Complete resistance, developed in the case of an incompatible interaction, is usually governed by the gene-for-gene system, and also called race-specific resistance. Much research has focused on this form of resistance which is generally inherited as a monogenic trait and is determined by the concomitant presence of a resistance (*R*) gene in the plant and the corresponding avirulence (avr) gene in the pathogen. Mechanistically, specific resistance relies on the recognition of *avr* pathogen factors by plant *R* gene products and the elicitation of local defense responses, often associated with a rapid programmed cell death, called the hypersensitive response (HR). A variety of *R* genes have been cloned from model and crop plants, and many *avr* genes have been characterized from bacteria, fungi and oomycetes[10]. Interestingly, although *R* genes confer resistance to diverse pathogens, their products share structural similarities suggesting the conservation of some signalling events in plant defense [10].

In contrast, the so-called partial resistance is quantitative, presumably non race-specific, and polygenic [11]–[13]. It limits the extent of disease caused by virulent pathogens and constitutes an additional layer of resistance in the absence of R function, during compatible interactions. The genetics of partial resistance has been characterized in many crop plants, such as rice and barley [14], [15] but remains poorly understood in Arabidopsis. One way to increase our knowledge in this field is a genetic study of the quantitative variation in resistance to virulent pathogens.

Although QTL analyses are increasingly used to study complex traits in Arabidopsis, such as developmental and yield traits [16], [17], only a few studies have investigated the genetic bases of quantitative variation in resistance and susceptibility to pathogens [18]–[22]. In most of these studies one or two major QTLs and a few minor loci were identified. In one study investigating plant susceptibility to the fungus *Botrytis cinerea*, multiple small-to-medium effect QTLs were identified [20], suggesting that multiple mechanisms are involved in susceptibility, or that a large number of polymorphic loci exert an effect on a particular mechanism. In some cases, genes underlying the QTL have been identified. For example, the major QTL for resistance to *Plectosphaerella cucumerina* was demonstrated to correspond to the ERECTA gene [22] which is implicated in plant development and also contributes to resistance to *Ralstonia solanacearum* [19].

The Arabidopsis-*Pseudomonas* interaction is a model pathosystem [23], [24] and has largely contributed to a better understanding of pathogen recognition in plants, pathogen virulence and avirulence determinants, host susceptibility and signal transduction pathways controlling plant defense responses [25]. In a previous study, analysis of natural variation in tolerance indicated that it behaves as a quantitative trait [26]. Tolerance, which can be defined as the ability of the host to endure the presence of the pathogen and to express less severe disease symptoms or less damage [27], differs from resistance in that symptom formation is uncoupled from pathogen growth [28]. When the genetic basis of variation of this trait was analysed in the Arabidopsis accessions Col-0 and No-0, only two minor loci for symptom severity and no QTL for bacterial colonization could be identified [21], although bacterial growth is a key quantitative component of the compatible interaction between Arabidopsis and the endophytic bacterial pathogen *Pst*.

In this paper, we use QTL analysis to investigate the genetic basis of partial resistance to *Pst* using the virulent strain DC3000. Natural variation in Arabidopsis for partial resistance to *Pst* allowed us to identify parental lines exhibiting significant differences in this trait, and to choose a RIL population derived from crosses between the accessions Bay-0 and Shahdara for detailed genetic analysis. Quantitative evaluation of this RIL population after infection with *Pst* DC3000 showed that partial resistance to *Pst* is controlled by two major and two minor QTLs. Using the heterogeneous inbred family strategy (HIF) [29], the two major QTLs were validated. To investigate whether these two major loci could influence known signalling pathways, the expression of marker defense genes was analyzed in HIFs, revealing an influence of the *PRP-Ps2* locus on the signalling pathway involving salicylic acid.

## Results

### Natural variation for *Pst* resistance in Arabidopsis

In order to investigate natural variation of partial resistance to *Pst* in *Arabidopsis thaliana*, 27 Arabidopsis accessions were tested for their response to the virulent *Pst* strain DC3000. The accessions correspond to a core-collection composed of 16 accessions which are estimated to represent most of the variation present within the species *Arabidopsis thaliana* [30], plus other parental accessions of RIL populations that are publicly-available or under construction (http://www.inra.fr/vast/RILs.htm).

**Figure 1:**
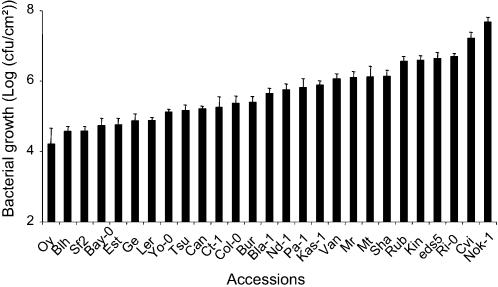
Natural variation of partial resistance to *Pseudomonas syringae* pv. *tomato* DC3000 among Arabidopsis accessions. *In planta* bacterial growth was assessed three days post inoculation with a bacterial suspension adjusted to 10^5^ cfu/mL. Means and standard errors were calculated from bacterial densities in at least 4 plants.

Plant resistance was evaluated by the measurement of bacterial *in planta* growth 3 days after leaf-infiltration of a bacterial suspension. The chosen inoculation procedure circumvents some layers of resistance operating in a natural infection process, but it is proven to be highly reproducible and allows the quantitative evaluation of resistance and susceptibility. As shown in Figure 1, bacterial growth varied continuously over a range of four orders of magnitude in the different accessions. This result indicates that partial resistance to *Pst* is a quantitative trait, suggesting that it may be under polygenic control.

Among the parental accessions showing very contrasting phenotypes, L*er* and Cvi seemed to be good candidates for further QTL analysis, since a well characterised L*er*×Cvi RIL population exists [31], [32]. However, the analysis of selected RILs from this population revealed, in accordance with published data [33] that 25% of the RILs are very early-flowering even under short day conditions. This was a major obstacle for the use of this population, because the evaluation of resistance is performed on fully expanded rosette leaves of non flowering plants. As the accessions Bayreuth (Bay-0) and Shahdara (Sha) also exhibited contrasting phenotypes, a F_6_ RIL population of 420 lines derived from these lines was chosen for QTL analysis [34]. Evaluation of resistance of reciprocal F_1_ hybrids between Bay-0 and Shahdara revealed that bacterial densities in F_1_ plants were similar to that evaluated in the parental line Shahdara, and higher than that in Bay-0 (Supplementary Figure 1).

### QTL analysis identifies two major and two minor loci for partial resistance

To identify loci responsible for the genetic differences in partial resistance to *Pst* between Bay-0 and Shahdara, three independent experiments, with two blocks in a complete randomized design, were performed on 165 RILs in greenhouse conditions, and one experiment was performed using 370 RILs under growth chamber conditions (Table 1). A total number of 1730 plants were evaluated for their response to *Pst*, using generally 14 plants per RIL.

**Table 1:**
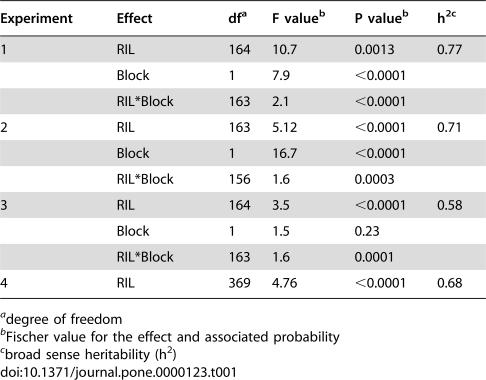
The influence of genotype on partial resistance to *Pseudomonas syringae* pv. *tomato* DC3000.

Experiment	Effect	df^a^	F value^b^	P value^b^	h^2^ c
1	RIL	164	10.7	0.0013	0.77
	Block	1	7.9	<0.0001	
	RIL*Block	163	2.1	<0.0001	
2	RIL	163	5.12	<0.0001	0.71
	Block	1	16.7	<0.0001	
	RIL*Block	156	1.6	0.0003	
3	RIL	164	3.5	<0.0001	0.58
	Block	1	1.5	0.23	
	RIL*Block	163	1.6	0.0001	
4	RIL	369	4.76	<0.0001	0.68

a degree of freedom

b Fischer value for the effect and associated probability

c broad sense heritability (h^2^)

**Figure 2:**
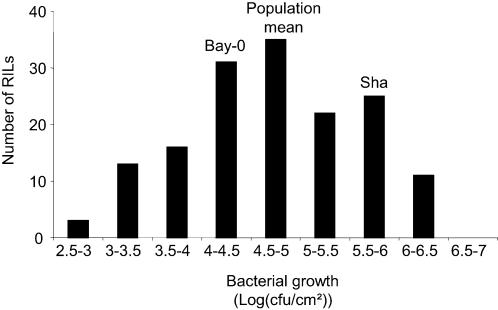
Distribution of bacterial growth values in the Bay-0×Shahdara recombinant inbred line (RIL) population. The frequency histogram shows the range of *in planta* bacterial populations observed in RILs three days post inoculation with *Pseudomonas syringae* pv. *tomato* DC3000 in one of the greenhouse experiments. The values obtained for the parental accessions, Bay-0 and Shahdara (Sha), and the genetic mean of the population are indicated.

Distributions of the RILs, according to bacterial populations measured *in planta* (log(cfu/cm^2^)), showed a continuous variation, suggesting a quantitative and polygenic control of the response (Figure 2). The parental accessions Bay-0 and Shahdara showed, as expected, different levels of bacterial growth. Moreover, transgressive segregation was observed in the RILs. Data obtained from the four experiments performed under different environmental conditions showed significant correlations (Table S1), and within each experiment, the randomized blocks were significantly correlated with R^2^ values varying from 0.57 to 0.76 (data not shown). Variance analysis of the phenotypic data showed that differences among RILs were highly significant (Table 1). The block effect, except for one of the experiments (experiment 3), and the RIL/block interaction were also significant for all the experiments performed in the greenhouse. Two plants for each RIL were evaluated for their response to *Pst* in each block, but no significant plant effects could be detected (data not shown). Broad-sense heritabilities calculated from the different experiments, ranged from 0.58 to 0.77, indicating that most of the phenotypic variation appeared to be genetically determined (Table 1).

**Figure 3:**
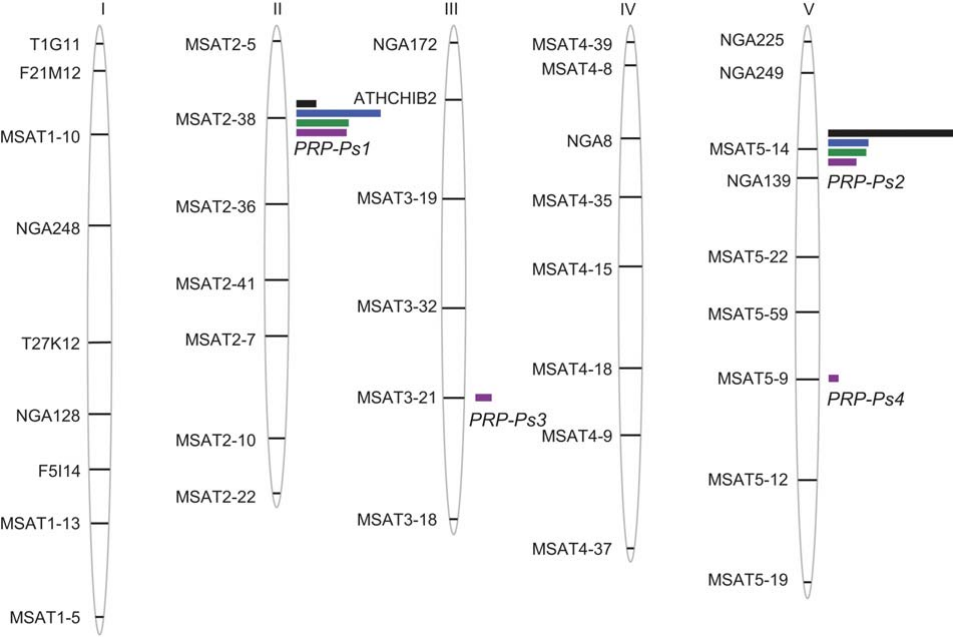
Arabidopsis QTLs controlling partial resistance to *Pseudomonas syringae* pv. *tomato* DC3000 in the Bay-0×Shahdara recombinant inbred line population. The detected QTLs are represented by bars located at the closest marker position (black, experiment 1; blue, experiment 2; green, experiment 3; purple, experiment 4) on the Bay-0×Shahdara genetic map [34]. The length of the bar is proportional to the QTL effect (R^2^).

For QTL detection, only composite interval mapping (CIM) results are presented for each experiment, because as compared to other methods, they provide a more accurate estimation of R^2^ values (phenotypic variances explained by the QTL) and additive effects [35]. Besides, the results of QTL detection obtained for each block of the experiments were the same as those obtained with the adjusted means on blocks (data not shown). Two major QTLs for partial resistance to *Pst,* *PRP-Ps1* (*Partial Resistance to* *Pathogen* - *Pseudomonas 1*) and *PRP-Ps2*, were detected in all experiments (Figure 3). *PRP-Ps1* was localized on chromosome 2 and explained more than 1/4 of the phenotypic variance (up to 42%) except in experiment 1 where it explained only 9%. *PRP-Ps2* usually explained less than 20% of the phenotypic variance, whereas its effect was much stronger in experiment 1 where it explained 62% (Table 2). The allelic additive effects of these QTLs are in the same direction, the Bay-0 allele increasing partial resistance compared to the Shahdara allele. In experiment 4, performed under growth chamber conditions, additional minor QTLs were detected on chromosomes III (R^2^ = 8%) and V (R^2^ = 5%). In contrast to the major loci, the Shahdara allele at these minor QTLs improves partial resistance to *Pst*. These observations might explain at least in part, transgressions above the Shahdara phenotypic value and below the Bay-0 phenotypic value in the RIL population (Figure 2).

**Table 2:**
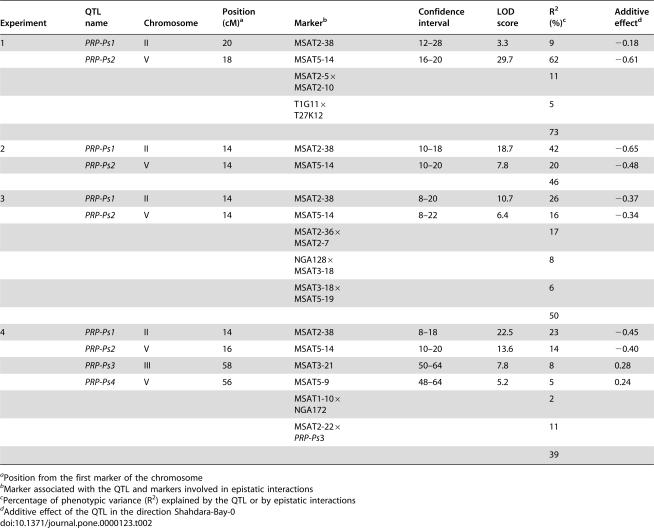
Arabidopsis QTLs controlling partial resistance to *Pseudomonas syringae* pv. *tomato* DC3000 in the Bay-0×Shahdara recombinant inbred line population.

Experiment	QTL name	Chromosome	Position (cM)^a^	Marker^b^	Confidence interval	LOD score	R^2^ (%)^c^	Additive effect^d^
1	*PRP-Ps1*	II	20	MSAT2-38	12–28	3.3	9	−0.18
	*PRP-Ps2*	V	18	MSAT5-14	16–20	29.7	62	−0.61
				MSAT2-5×MSAT2-10			11	
				T1G11×T27K12			5	
							73	
2	*PRP-Ps1*	II	14	MSAT2-38	10–18	18.7	42	−0.65
	*PRP-Ps2*	V	14	MSAT5-14	10–20	7.8	20	−0.48
							46	
3	*PRP-Ps1*	II	14	MSAT2-38	8–20	10.7	26	−0.37
	*PRP-Ps2*	V	14	MSAT5-14	8–22	6.4	16	−0.34
				MSAT2-36×MSAT2-7			17	
				NGA128×MSAT3-18			8	
				MSAT3-18×MSAT5-19			6	
							50	
4	*PRP-Ps1*	II	14	MSAT2-38	8–18	22.5	23	−0.45
	*PRP-Ps2*	V	16	MSAT5-14	10–20	13.6	14	−0.40
	*PRP-Ps3*	III	58	MSAT3-21	50–64	7.8	8	0.28
	*PRP-Ps4*	V	56	MSAT5-9	48–64	5.2	5	0.24
				MSAT1-10×NGA172			2	
				MSAT2-22×*PRP-Ps*3			11	
							39	

a Position from the first marker of the chromosome

b Marker associated with the QTL and markers involved in epistatic interactions

c Percentage of phenotypic variance (R^2^) explained by the QTL or by epistatic interactions

d Additive effect of the QTL in the direction Shahdara-Bay-0

Testing of the 703 possible pairwise interactions revealed highly significant digenic epistatic interactions (Table 2). Most of them occurred between markers with no additive effects and only a few of them were detected between a QTL (or close to the QTL) and a marker of the genetic background.

The total phenotypic variance explained by QTLs with additive effects for partial resistance to *Pst* ranged from 37% to 67% (data not shown), and increased to 39% to 73% when epistatic interactions were included (Table 2).

### Confirmation of the major QTLs *PRP-Ps1* and *PRP-Ps2* in near isogenic lines

**Figure 4:**
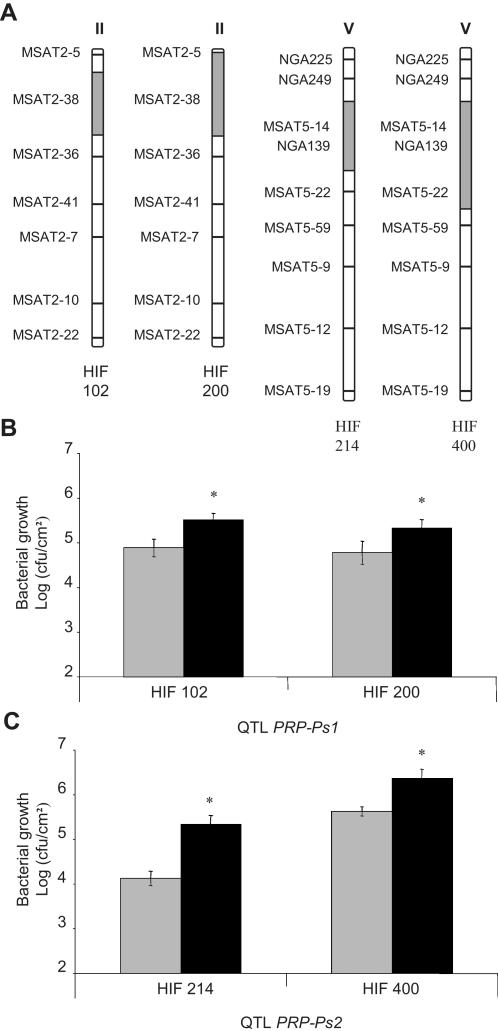
Validation of the major QTLs for partial resistance to *Pseudomonas syringae* pv. *tomato* DC3000 with heterogeneous inbred families (HIFs). **A** HIF 102 segregates around MSAT2-38 and HIF 200 segregates for a region of chromosome II around markers MSAT2-5 and MSAT2-38. HIF 214 segregates for a region of chromosome V around MSAT5-14 and NGA139 and HIF 400 segregates around MSAT5-14, NGA139 and MSAT5-22. Regions for which the HIFs segregate are indicated in hatched boxes and the white regions of the chromosomes represent a mix of both parental genomes Bay-0 and Shahdara. **B** and **C** *In planta* bacterial growth in HIFs fixed for the Bay (grey bar) or Sha (black bar) allele of *PRP-Ps1* (**B**) or *PRP-Ps2* (**C**). Each value is the average of measurement of *in planta* bacterial growth in at least 8 plants (means and standard errors) from one experiment. Three independent experiments were performed and showed similar results. Asterisks show significant difference in partial resistance (P<0.05).

To confirm the effects of the major QTLs *PRP-Ps1* and *PRP-Ps2* in near isogenic lines (NILs), RILs still segregating for the region of interest (so-called heterogeneous inbred families (HIFs)) were identified. Two sets of HIFs, segregating for the *PRP-Ps1* locus surrounding marker MSAT2-38, and two sets segregating for the *PRP-Ps2* locus surrounding marker MSAT5-14, were evaluated for partial resistance to *Pst* (Figure 4A). Each of the chosen RILs displays a different genetic background, a mix of both parental genomes Bay-0 and Shahdara.

As shown in Figure 4B and 4C, all the HIF lines carrying the Bay allele at *PRP-Ps1* or *PRP-Ps2* showed significantly lower bacterial colonization than the corresponding NILs carrying the Sha alleles. *In planta* bacterial growth was between 5 to 10 fold lower when the Bay-0 alleles were present. These results confirmed the quantitative contribution of both loci to partial *Pst* resistance and validated the major QTLs, *PRP-Ps1* and *PRP-Ps2*.

### *PRP-Ps2* influences SA-dependent gene expression

Genetic analyses revealed that the balance between signalling components such as ethylene (ET), jasmonates (JA) and salicylic acid (SA) is crucial to modulate and adapt the various defense mechanisms to a given pathogen [36]. While JA and ET are important positive regulators in the resistance to necrotrophic pathogens, SA has been demonstrated to play a central role in resistance to biotrophs [36], [37]. Partial resistance to *Pst* relies to a large part on SA dependent defense responses and is negatively regulated by the JA pathway.

**Figure 5:**
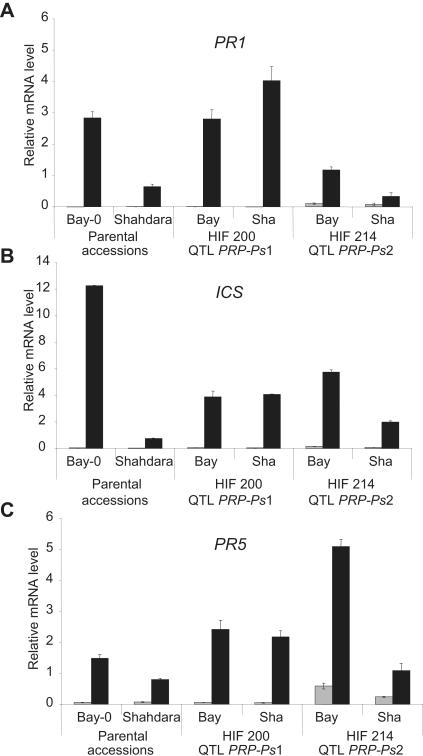
Expression analysis of the defense marker genes *PR1* (A), *ICS* (B) and *PR5* (C) in HIFs and parental accessions. The transcript levels were determined by Q-RT-PCR with cDNA generated from leaves before (grey) and after (black) inoculation with *Pst* DC3000 at 5.10^5^ cfu/mL. The expression value of the individual genes was normalized by using the expression level of *β-Tubulin4* as an internal standard. Mean mRNA levels of three plants are shown with corresponding standard errors. Similar results were obtained from two biological experiments.

We therefore analysed to what extent *PRP-Ps1* and *PRP-Ps2* influenced signalling through these pathways. The expression of marker genes of SA and JA/ET signalling was investigated in the lines HIF200 and HIF214 segregating at the *PRP-Ps1* or *PRP-Ps2* locus, respectively. For SA-dependent signalling, the expression of the defense genes *PR1* (*Pathogenesis Related 1*), *PR5* (*Pathogenesis-Related 5*) and *ICS* (*IsoChorismate Synthase*) was analysed in parental ecotypes and in HIFs before and 24 h after infection with virulent *Pst* (Figure 5) [38], [39]. As expected, all three marker genes showed stronger pathogen-responsive expression in the more resistant Bay-0 accession than in Shadhara (Figure 5). Interestingly, they were also expressed at significantly higher levels in HIF214 plants that carry the Bay allele of *PRP-Ps2*, than in HIF214 plants carrying the Sha allele. Thus, the differences observed between the HIFs for *in planta* bacterial growth at the *PRP-Ps2* locus can be correlated with those observed for expression of SA-dependent genes, suggesting an implication of *PRP-Ps2* in the activation of SA dependent responses. In contrast, there was no significant difference in expression levels of SA marker genes when HIF200 plants were analysed, suggesting that the *PRP-Ps1* locus does not influence SA-dependent gene expression.

For JA- and ET-dependent signal transduction pathways, the expression of *PR3* (*Pathogenesis-Related 3*) and *VSP* (*Vegetative Storage Protein*) was analysed [38], [40]. Although these genes were induced upon inoculation, no correlation between resistance level and gene induction level could be observed when parental accessions or the HIFs were compared (data not shown).

## Discussion

In natural populations there is a prevalence of low level, partial resistance that often prevents pathogens from reaching seriously damaging levels. Several studies have identified major genes controlling partial resistance in various crops [41]–[43]. However, the molecular mechanisms underlying these loci were not identified.

In the model plant Arabidopsis, while the characterization of simply inherited *R* genes mediating strain-specific recognition of pathogens and complete resistance has been intensively investigated, the mechanisms that control partial resistance are poorly understood. Evidence has however been obtained that the different types of resistance already described (nonhost, complete, partial) have several features in common. For example, genes necessary for *R*-gene mediated resistance are also involved in non-host and partial resistances in Arabidopsis. For example, *eds1,* *pad4* and *sag101* mutants, which are impaired in *R*-gene dependent resistance, show enhanced susceptibility to the virulent bacterium *Pst* and allow invasive growth of non-host powdery mildew isolates [2], [44]–[46]. In addition, whole genome gene expression analysis [47] and the study of the expression of individual genes [48], indicate similar transcriptional changes in compatible and incompatible interactions with *Pst*, with most differences in the defense transcriptome being due to the kinetics and the amplitude of the response. In addition, defense mechanisms involved in partial, complete and nonhost resistances, are modulated by many type III effector proteins (TTEs) delivered by bacteria during infection [49], [50]. Interestingly, the outcome of the interaction between effectors and defense mechanisms depends not only on the nature of the effector but also on the kinetics of its delivery by the bacteria [51]. In conclusion, these findings point to a complex network of regulators and regulatory mechanisms operating for the different forms of resistance in Arabidopsis. Although genetic analyses have clearly identified several *R* genes and some *EDS* genes acting in complete resistance pathways and affecting other types of resistance, to our knowledge and with the exception of the work of Kover already mentioned [21], no such a direct genetic approach has been conducted for partial resistance to the model pathogen *Pst* in Arabidopsis, which should reveal major actors of this resistance.

The most important finding of this study was the discovery in Arabidopsis of two major loci governing variation in partial resistance to *Pst*, in the Bay-0×Shahdara RIL population. In addition, the validation of these loci opens the way for their fine mapping and their cloning, which will improve our understanding of the molecular mechanisms underlying partial resistance.

### PRP-Ps1 and PRP-Ps2, two major traits controlling partial resistance to Pseudomonas

Two major QTLs and two minor loci controlling partial resistance to *Pst* have been identified through our quantitative analysis. For the two major QTLs, the Bay-0 allele enhances the resistance to *Pst*. On the contrary, for the two minor QTLs, *PRP-Ps3* and *PRP-Ps4*, the Shahdara allele enhances the resistance to *Pst*. Alleles from the susceptible parent that enhance disease resistance were already reported for several host-pathogen interactions [52]–[55]. This may also explain transgression towards susceptibility in the RIL population.

Highly significant correlations between experiments validate reproducibility of the phenotypic evaluations. The homogeneous behaviour of RILs also contributed to confer high heritabilities, indicating that the variations observed are mostly genetically controlled and that the estimation of partial resistance by phenotypic evaluations is reliable. However, the detected QTLs explained from 39% to 73% of the phenotypic variance, and comparison with the narrow sense heritabilities (from 0.58 to 0.77) suggests that not all the genetic variance is explained by these QTLs. This may result either i) from the choice of the significance threshold (2.7) which could have prevented the detection of minor QTLs, or ii) from the underestimation of the QTL contribution. Another explanation could be the involvement of the epistatic interactions detected in our study and which can potentially have an important contribution in genetic variation in complex traits [56].

Strong interaction with the environment is one of the hallmarks of quantitative traits and it is not uncommon that the magnitude of QTLs varies with changing environmental conditions or even, that QTLs are only expressed in a given environment and not in others [57], [58]. Environmental effects on epistatic loci are even more pronounced [59], [60]. Therefore plant resistance and susceptibility are strongly influenced by environmental conditions, and must be studied in controlled experimental setups. The fact that, in another study investigating natural variation in tolerance of Arabidopsis to *Pst*, different resistance levels were found for several accessions [26], may be due to such environmental effects. Although environment-genotype interactions were not evaluated in our study, we detected significant “experiment” and “block” effects (Table 1) suggesting that environmental conditions influence partial resistance to *Pst*. In addition, the relative contributions of the major QTLs, *PRP-Ps1* and *PRP-Ps2,* to the overall phenotypic values showed some variation between experiment 1 and experiments 2, 3 and 4, and the detected epistatic interactions were not consistent for all experiments. However, despite the apparent effects of the environment on partial resistance, the two major QTLs, *PRP-Ps1* and *PRP-Ps2*, were detected in four independent experiments whatever the environmental conditions, and could be confirmed in NILs using the powerful HIF strategy [29]. The fact that the effects of *PRP-Ps1* and *PRP-Ps2* can be detected in segregating populations opens the way for their fine-mapping and their molecular cloning. Knowing the molecular identity of *PRP-Ps1* and *PRP-Ps2* will not only give new, fundamental insights into partial resistance, but also allow to study on a molecular level, the plasticity of partial resistance.

### Candidate loci

Two major QTLs, and two minor loci accounting for about one half of the phenotypic variation have been identified through our quantitative analysis, suggesting that a few major mechanisms are controlling partial resistance to *Pst*. This is surprisingly similar to complete resistance in which the genetics of the plant-pathogen interaction is under the control of one or a few major genes. Consequently, it would be interesting to investigate whether putative disease resistance proteins, or other candidate genes related to different types of resistance (as described earlier), are found at the genomic location of the QTLs identified in this study. However, since no pathogen resistance QTL has been cloned as such and only a few genes acting as resistance QTL in defined interactions have been identified, the molecular nature of such genes remains elusive.

Partial resistance QTLs frequently co-localize with known *R* gene loci and it is assumed that many QTLs correspond to weak or defeated *R*-genes with, in most cases, the canonical NB-LRR domains[61]. Since resistance genes are highly polymorphic within species and populations [62], such QTLs may correspond to allelic variants of qualitative resistance genes with intermediate phenotypes [63]. For example, the rice *Xa21D* gene confers partial resistance to bacterial blight, while other *Xa21* alleles confer complete resistance [64]. In barley, clustering of major resistance gene and resistance QTL has also been reported [65]. *PRP-Ps2*, *PRP-Ps3* and *PRP-Ps4* co-localize with regions containing hypothetical NB-LRR resistance genes (http://niblrrs.ucdavis.edu/At-RGenes) [66], making them potential candidate genes. Thus, within the confidence interval defined for the QTL *PRP-Ps4* on chromosome V, is located a cluster of resistance genes, and among them the *R* gene *RPS4*. In addition, *PRP-Ps2* and *PRP-Ps3* co-localize with a resistance QTL to powdery mildew, *RPW11* [18], and with a QTL detected for tolerance to *Pst* associated with symptom severity [21], respectively.

In addition to a role in pathogen perception, partial resistance QTLs may also operate in signal transduction. Multiple signalling elements of resistance have been identified by genetic means and a central role of SA production and signal transduction is well established [67]. However, none of the identified genes nor *eds*, co-localize with the *PRP-Ps* QTLs.

Partial resistance QTLs might also correspond to genes involved in constitutive defense responses. The cell wall is generally believed to be an efficient physical barrier against microbial attack. Alterations in cell wall structure could therefore result in altered partial resistance. QTL analysis employing the Bay-Sha population has detected multiple loci influencing cell wall structure [68] and some of them co-localize to *PRP-Ps* QTLs. *PRP-Ps1* co-localizes with the ARH-1 QTL for the Ara-Rha ratio reflecting difference in RGI (rhamnogalacturonan I) structure and *PRP-Ps3* and *PRP-Ps4* co-localize with two minor QTLs, HLD-3 and HLD-4 (HLD for dark-grown hypocotyl length) for hypocotyl length reflecting cell elongation.

In summary, a number of genes putatively associated with the plant defense system, co-localize with some of the *PRP-Ps* loci. However, the confidence intervals found for these QTLs involve rather large genomic regions, which need to be reduced using HIFs to define candidate genes more precisely.

### Gene expression/pathways controlled by PRP-Ps1 and PRP-Ps2

The central role of SA-dependent defense responses in resistance to *Pst* is well established [36]. Mutants affected in SA production or signal transduction show reduced resistance to *Pst* [39]. The finding that the resistance level in HIFs differing in the *PRP-Ps2* locus, correlates with the expression of marker genes of the SA response is therefore particularly interesting. It indicates that the *PRP-Ps2* locus might be involved in the activation of inducible defence responses associated with partial resistance, but it raises the question at what level *PRP-Ps2* is acting; in SA production, in SA signalling or up-stream of SA? Quantification of SA levels and responsiveness of HIFs to SA application should provide answers to this question. *PRP-Ps1*, in contrast, seems to act independently of SA. At least SA pathway marker genes show no differential expression in an HIF segregating for this locus. Therefore, *PRP-Ps1* may act in constitutively expressed defense responses or in SA-independent pathways necessary for complete resistance. Such pathways have been revealed by mutant and transcriptome analysis [38], [69] and their elements are beginning to be identified [70].

In this context, an interesting recent study aimed at identifying loci controlling transcriptome variation by global eQTL analysis [71]. Categorizing eQTLs has the potential to enable reverse genetics for the identification of genes controlling quantitative traits and may also help to enhance the rate of QTL cloning. Interestingly in this analysis, several eQTLs detected on chromosomes II, III and V are related to networks involved in plant-pathogen responses, suggesting that they may influence variation in plant-pathogen interactions in the Bay-0×Sha population. By mapping QTLs for partial *Pst* resistance in the same RIL population that has been used for global gene expression analysis, the relationship between partial resistance to *Pst* and genetic architecture of eQTLs could be evaluated. More generally, combining QTL mapping with whole genome expression analysis to identify transcripts corresponding to QTLs will give a more complete picture of the complex genetic architecture of quantitative traits [57].

In the future, molecular cloning of the QTLs identified in our study will certainly help to understand the molecular basis of quantitative variation of partial resistance to *Pseudomonas syringae* in Arabidopsis, a model pathosystem in the plant pathology field.

## Methods

### Plant material and growth conditions

The Bay-0×Shahdara RIL population and the heterogeneous inbred families (HIFs) derived from the RIL population were developed by Loudet *et al.* [34] and unpublished. A complete description of the RIL population is available at http://dbsgap.versailles.inra.fr/vnat/Fichier_collection/Rech_rils_pop.php. HIFs were obtained as previously described [72]. The reciprocal F_1_ hybrids derived from the cross between Bay-0 and Shahdara (F_1_ BS) and between Shahdara and Bay-0 (F_1_ SB) were generated in our laboratory. Plants were grown in a growth chamber at 22°C, with a 9h light period and a light intensity of 190 µmol/m^2^/s. All experiments were performed with 4 to 5 week-old plants.

### Bacterial strains, plant inoculation procedure and bacterial growth measurement

*Pseudomonas syringae* pv. *tomato* (*Pst*) DC3000 strain was grown at 29°C on KingB's medium supplemented with 50 µg/mL of rifampicin [73]. For the determination of *in planta* bacterial growth, we used an inoculum of 4.10^4^ cfu/mL. For gene expression analysis, we used an inoculum of 5.10^5^ cfu/mL. Plant inoculations and *in planta* bacterial growth analysis were performed essentially as described previously with two to four plants per RIL and per experiment [74]. In order to analyze *in planta* bacterial densities in hundreds of plants in parallel, leaf discs were harvested in 96 deep-well microtiter plates. Bacteria were extracted by addition of 0.2% Silwet L-77 and shaking for 30 minutes. Subsequent serial dilutions and depositions on culture medium were performed by using microtiter plates and multichannel pipettes.

### Experimental design for Bay-0xShahdara RIL population and HIFs

The RIL population was evaluated by performing 4 experiments under different conditions. Three experiments were conducted with 165 RILs of the population under greenhouse conditions and another one with 370 RILs under growth chamber conditions. For the three experiments performed in greenhouse, each RIL was evaluated in a complete randomized block design. Two plants of each RIL per block with two blocks in each experiment were evaluated for partial resistance to *Pst*. For the other experiment in growth chamber, complete randomized design of RILs with two plants for each RIL was used.

HIFs were also evaluated for partial resistance to *Pst* by *in planta* bacterial growth analysis. At least, 32 plants of each HIF were tested in two to three independent experiments.

In the case of the evaluations of the F_1_ hybrids, 16 plants were tested for each cross.

### Statistical analyses

Data was analyzed for each block and each experiment. Adjusted means of disease scores (lsmeans) of RILs in blocks were estimated from variance analysis (ANOVA). Phenotypic correlations among the blocks, the experiments, and the variables were calculated. When replicates were available for an experiment, broad sense heritabilities (h^2^) were estimated from the mean square (MS) of ANOVA using the formula adapted from Gallais [75]:
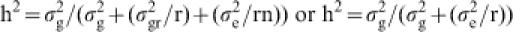
where σ^2^_g_ is the genetic variance (MSg-MSgr)/rn, σ^2^_gr_ the genotype*block interaction (MSgr-MSe)/n, σ^2^_e_ the environmental variance (MSe), n the number of plants and r the number of replicates.

Data analyses were performed with Statistical Analysis System (SAS) software (SAS Institute Inc., North Carolina, USA). Variance analysis of *in planta* bacterial growth data was performed using PROC GLM of SAS with randomized effects.

### QTL detection

The QTL was detected on the lsmeans of *in planta* bacterial growth data for each experiment, and on the means of the *in planta* bacterial growth data of each block (when the block effect was highly significant, P<0.01). Variance analysis (LR), Interval mapping (IM) and composite interval mapping (CIM) were performed with the QTL Cartographer software Version 1.17 (Basten et al, North Carolina State University, USA) for each trait. Interval mapping (IM) and composite interval mapping (CIM) were also performed with PLABQTL Version 1.2 (Utz and Melchinger, University of Hohenheim, Germany). After performing 1000 permutations with ANOVA, a LOD threshold of 2.7 was used to declare a putative QTL significant. For CIM, 2 to 4 of the most informative markers per trait were chosen as cofactors. For each trait, a multiway ANOVA was performed with molecular markers near the QTL peaks to estimate the total percentage of phenotypic variation (R^2^) explained by the significant QTLs. QTLs were named *PRP-Ps* for Partial Resistance to Pathogen - *Pst*. Confidence intervals of the detected QTLs were estimated from the PLABQTL software.

In addition to additive effects, digenic epistasis was tested with a two-factor ANOVA model with an interaction between pairs of markers. With the PROC GLM of SAS software, 703 interaction tests were performed and a significance level of *P*<0.001 (0.7 false positive) was chosen for detecting digenic epistasis. Global R^2^ was estimated with full ANOVA including all additive effects and digenic epistatic effects.

### RNA extraction and Q-RT-PCR analysis

Material for RNA analysis was ground in liquid nitrogen and total RNA was isolated using the Macherey-Nagel Nucleospin RNA plant kit (Macherey-Nagel, Hoerdt, France) according to the manufacturer's recommendations. Reverse transcription was performed with 1 µg of total RNA using the superscript reverse transcriptase II (Invitrogen, Carlsbad, CA, USA). Quantitative PCR was run on a Lightcycler system (Roche Diagnostics, Meylan, France) according to the manufacturer's recommendations with the following conditions: 1 cycle: 9 min at 95°C for; 45 cycles: 5 sec at 95°C, 10 sec at 65°C and 20 sec at 72°C. *β-Tubulin4* was used as an internal standard. The primer sets used are for *PR1* (locus At2g14610, forward primer: GGAGCTACGCAGAACAACTAAGA, reverse primer: CCCACGAGGATCATAGTTGCAACTGA), for *PR5* (locus At1g75040, forward primer: CGGTACAAGTGAAGGTGCTCGTT, reverse primer: GCCTCGTAGATG GTTACAATGTCA), and for *ICS* (At1g74710, forward primer: GCCGTCTCTGAAC TCAAATCTCAA, reverse primer: GTTACGAGCAAGAACAACCTTGTT). Specificity of the amplifications was verified by melting curve analysis. Efficiency of the amplification was verified by the analysis of standard curves.

## Supporting Information

Figure S1 Analysis of the F1 progeny of reciprocal crosses between Bay-0 and Shahdara for partial resistance to *Pseudomonas syringae* pv. *tomato* DC3000. *In planta* bacterial growth was assessed three days post inoculation in the F1 generation of reciprocal crosses between Bay-0 and Shahdara. Each value is the average of *in planta* bacterial growth of at least 4 plants (means and standard errors).
(0.62 MB EPS)

Table S1 Phenotypic correlations among experiments
(0.04 MB DOC)
