# Supplementary material for: Natural Variation in Partial Resistance to Pseudomonas syringae Is Controlled by Two Major QTLs in Arabidopsis thaliana
Source: PLoS One. 2006 Dec 27;1(1):e123. doi: 10.1371/journal.pone.0000123 (PMC1762404; doi:10.1371/journal.pone.0000123)
Supplement: Table S1 — Phenotypic correlations among experiments (0.04 MB DOC) [file pone.0000123.s002.doc]

**Table S1**. Phenotypic correlations among experiments

|  | Experiment 2 | Experiment 3 | Experiment 4 |
| --- | --- | --- | --- |
| Experiment 1 | 0.59* | 0.53* | 0.36* |
| Experiment 2 |  | 0.63* | 0.63* |
| Experiment 3 |  |  | 0.56* |

* Significantly different from 0 at the 0.001% level
